# Soft Nanocomposite Based Multi-point, Multi-directional Strain Mapping Sensor Using Anisotropic Electrical Impedance Tomography

**DOI:** 10.1038/srep39837

**Published:** 2017-01-25

**Authors:** Hyosang Lee, Donguk Kwon, Haedo Cho, Inkyu Park, Jung Kim

**Affiliations:** 1 Korea Advanced Institute of Science and Technology, 291 Daehak-ro, Yuseong-gu, Daejeon 34141, Republic of Korea

## Abstract

The practical utilization of soft nanocomposites as a strain mapping sensor in tactile sensors and artificial skins requires robustness for various contact conditions as well as low-cost fabrication process for large three dimensional surfaces. In this work, we propose a multi-point and multi-directional strain mapping sensor based on multiwall carbon nanotube (MWCNT)-silicone elastomer nanocomposites and anisotropic electrical impedance tomography (aEIT). Based on the anisotropic resistivity of the sensor, aEIT technique can reconstruct anisotropic resistivity distributions using electrodes around the sensor boundary. This strain mapping sensor successfully estimated stretch displacements (error of 0.54 ± 0.53 mm), surface normal forces (error of 0.61 ± 0.62 N), and multi-point contact locations (error of 1.88 ± 0.95 mm in 30 mm × 30 mm area for a planar shaped sensor and error of 4.80 ± 3.05 mm in 40 mm × 110 mm area for a three dimensional contoured sensor). In addition, the direction of lateral stretch was also identified by reconstructing anisotropic distributions of electrical resistivity. Finally, a soft human-machine interface device was demonstrated as a practical application of the developed sensor.

Recently, highly flexible and stretchable strain sensors that can function under large strains (>100%) with high gauge factors (>2) are in great demand for skin-like interactive applications to tactile sensing^1^,^2^,^3^, soft robots^4^,^5^, human motion detection^6^,^7^, and therapeutics^8^,^9^. Thus far, many studies on large strain sensing with high gauge factors have been reported based on elastomeric composites dispersed with conductive nanoparticles^10^,^11^, carbon nanotubes^12^,^13^,^14^,^15^,^16^,^17^,^18^, nanowires^1^,^2^,^19^,^20^,^21^, and graphenes^22^,^23^. Moreover, the advances of soft and stretchable conductors^13^,^24^,^25^,^26^,^27^,^28^ enabled the acquisition of tactile information from curved surfaces using arrays of stretchable electrodes^3^,^27^,^29^.

In order to apply stretchable strain sensors in practical applications, large area coverage of the sensor is considered as a challenging issue due to the difficulties in the manufacturing process and data acquisition^29^,^30^,^31^. Arrays of parallel stretchable electrodes and piezoresistive elastomeric composites have been commonly utilized to obtain the tactile information from large areas^3^,^6^,^18^,^29^. However, multi-directional tactile information requires additional stretchable electrodes in limited space, which increases the difficulties in the fabrication process. As an alternative solution, organic field-effect transistor (OFET) integrated on stretchable substrates was introduced as a mean to reduce the number of stretchable electrodes and to acquire multi-directional tactile information^32^,^33^. Although these works demonstrated large-area and stretchable electronics, repetitive and impulsive contact conditions can induce excessive mechanical stress to electronics and interconnections in practical applications such as human-like tactile sensing^3^,^4^,^34^,^35^,^36^,^37^,^38^ and biomedical applications^35^,^39^. These applications require tactile sensors that are highly sensitive with high spatial resolution for artificial skins (e.g. finger tips) as well as robust to repetitive and impulsive contact conditions. Furthermore, the capability of large and curved area coverage would be beneficial for the whole body tactile sensing^30^,^40^,^41^.

Stretchable conductive materials incorporated with electrical impedance tomography (EIT) can be an alternative solution to overcome aforementioned limitations^42^,^43^,^44^,^45^,^46^,^47^,^48^,^49^,^50^,^51^,^52^. Electrical impedance tomography is a technique that computes internal conductivity distribution from continuum conductive model using electrodes fabricated along the boundary of the conductive material^53^. Here, the electrodes are used to apply electrical currents and to measure electrical potentials. This technique has been used to demonstrate a stretchable and sensitive electronic skin covering on three dimensional contoured surfaces without any internal wiring using conductive fabric^43^,^44^,^50^,^51^,^52^, conductive rubber^46^,^47^,^48^, and conductive liquid^45^. In these literatures, no complex array of stretchable electrodes needs to be fabricated within the entire sensing area. In addition, the EIT based sensor could provide robustness to various contact conditions since the electrodes of the sensor could be located only along the boundary so that direct physical contact does not occur between the applied forces and the electrodes^49^. However, a detailed study on the identification of multi-directional strain mapping based on EIT has not been introduced so far because conventional EIT method could compute only homogeneous resistivity distribution of the conductive medium. In addition, the conductive medium exhibiting anisotropic resistivity when strain is induced was required.

Here, we demonstrate multi-point and multi-directional strain mapping sensors based on the nanocomposite of multiwall carbon nanotube (MWCNT)-silicone elastomer using anisotropic electrical impedance tomography (aEIT). The nanocomposite exhibits an anisotropic piezoresistivity due to the morphological changes of MWCNT network along the direction of the strain^20^,^54^,^55^. The aEIT method computes the multi-dimensional resistivity distribution within the nanocomposite, thereby the strain directions and contact locations on large curved surfaces can be accurately identified without using complicated arrays of flexible and stretchable electrodes fabricated along the entire sensor surface. We characterized the surface indentation force and location estimation performances and stretch displacement and direction estimation performances of the strain mapping sensor in detail. Finally we implemented a soft, three-dimensional human-machine interface device to control a robotic hand system.

## Results

### Device fabrication using MWCNT-silicone elastomer nanocomposite

**Figure 1:**
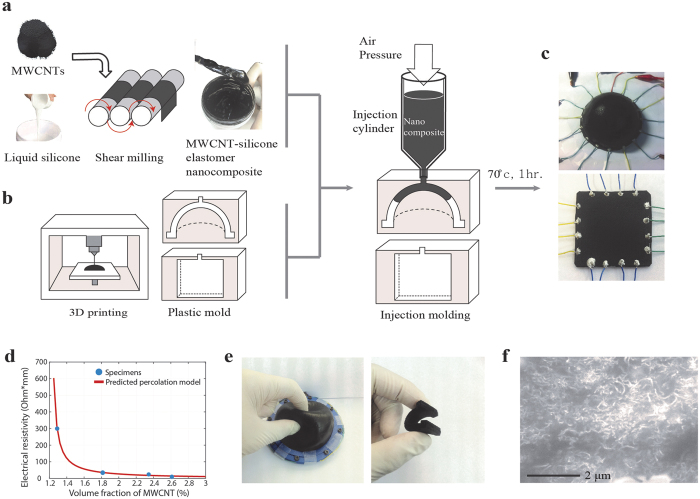
Fabrication of MWCNT-silicone elastomer nanocomposite based multidimensional strain sensor. Schematic illustration of (**a**) preparation of liquid MWCNT-silicone elastomer nanocomposite and (**b**) injection molding into 3D printed plastic mold. (**c**) Photography of fabricated hemisphere and square shaped strain sensor. (**d**) Electrical resistivity of MWCNT-silicone elastomer nanocomposite vs. volume fraction of the MWCNT. (**e**) Deformation of strain sensor under pinching and bending. (**f**) SEM images showing randomly dispersed MWCNTs in the silicone elastomer matrix.

As shown in Fig. 1a, uniform nanocomposite of multiwall carbon nanotubes (MWCNT) and liquid silicone (Ecoflex^®^) were dispersed by using shear milling, which enabled randomly oriented distribution of MWCNTs within the nanocomposites^56^. The MWCNTs form an electrically conductive network with controllable resistance by the volume fraction of the MWCNTs within the silicone elastomer. When the composites deform due to external forces, the electron transport within the conductive network is changed, thereby modulating the electrical resistance. This nanocomposite material can be easily fabricated into various two or three dimensional shapes by employing injection molding techniques^57^,^58^. Figure 1b shows the procedures to fabricate soft strain sensors by injection molding. Plastic molds were first fabricated in various shapes using three dimensional printing process. MWCNT-liquid silicone nanocomposite was filled into the injection mold using air pressure, followed by thermal curing in a convection oven at 70 °C for 1 hour. Hemispherical and rectangular parallelepiped specimens were fabricated by using this process (Fig. 1c). The conductivity of the specimens depended on the volume fraction of the MWCNT due to the percolation phenomenon (Fig. 1d) 59. The percolation threshold was estimated from the percolation model and resistivity of the specimens with different volume fractions of MWCNTs (see Supplementary Figure 1). The specimens showed highly soft characteristics and good recovery against repeated bending and squeezing (Fig. 1e). In addition, from the scanning electron microscopic (SEM) images, we could observe that the MWCNTs were uniformly dispersed in random directions within the silicone elastomer matrix (Fig. 1f). The electrical resistance of specimens with the 2.5(1.29) wt% (vol%) showed a standard deviation of 16.7% with respect to the average. (2633 ± 439 ohm-mm) (Supplementary Figure S2).

### Electro-mechanical characteristics of the MWCNT-silicone elastomer nanocomposite

**Figure 2:**
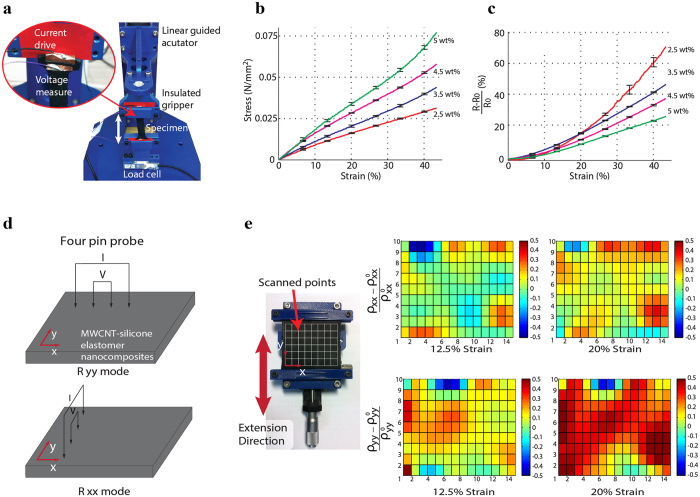
Piezoresistive characteristics of the MWCNT-silicone elastomer nanocomposites. (**a**) Photograph of the experimental set-up, Experimental results of (**b**) strain versus stress behavior and (**c**) strain versus resistance change, (**d**) schematics of measurement modes for resistivity along x and y directions, (**e**) experimental results of strain versus anisotropic resistivity distribution along x and y directions.

Tensile experiments were conducted to assess the piezoresistivity of the MWCNT-silicone elastomer nanocomposite. As shown in Fig. 2a, a custom-designed tensile tester was utilized to apply a tensile strain to the specimen while providing a constant current and measuring a voltage across the specimen simultaneously. Dog-bone shaped specimens were fabricated by injection molding. Dimensions and shapes of the specimen followed the JANNAF polymer testing standard^60^. Four specimens with different weight ratios of the MWCNT and elastomer were prepared (2.5 wt%, 3.5 wt%, 4.5 wt% and 5 wt%). Figure 2b shows the mechanical responses of each specimen. The graphs of the mechanical responses show typical characteristics of the elastomeric polymers. Mechanical stiffness of the composite increased from 76 kPa to 166 kPa by the increase of the weight ratio of the MWCNT in the nanocomposites (2.5 wt%, 3.5 wt%, 4.5 wt% and 5 wt%). The mechanical stiffness of 166 kPa is low enough to provide flexibility and softness for comfortable physical interaction with human users. The electrical resistances of the nanocomposites showed a linear relationship between voltage and current with 0% to 80% strains for all the specimens (Supplementary Figure S3). Figure 2c shows the electrical responses of the specimens to the applied strains. In this case, the resistance of the specimen increases with the extension in the range of 0–40% strain. The sensitivities of the specimens defined as (∆R/R_0_)/ε were estimated using linear regression for simplicity. From 0 to 40% strain, the sensitivities were 1.61, 1.12, 0.91, 0.67 for 2.5 wt%, 3.5 wt%, 4.5 wt%, 5 wt% of MWCNT within polymer matrix, respectively. To achieve relatively high sensitivity with low stiffness, weight percent of 2.5 wt% was selected to fabricate the sensing elements used in this work.

Investigating the anisotropic property in resistivity change against the external strain is necessary for the nanocomposite to achieve multi-dimensional strain sensing. For this reason, four point probe was used to measure the anisotropic resistivity distribution (Supplementary Figure S4). A linear guide stretched a square shaped specimen in y direction and four point probe contacted the surfaces of the specimen using 3 dimensional manipulator. The line of the four probes was aligned to the x axis to measure the resistivity change along y directions (ρ_yy_), and y axis to measure the resistivity change along x directions (ρ_xx_), respectively (Fig. 2d) 61. The relationship between resistance measured from four point probe and resistivity components are explained in Supplementary Note 2. The changes of resistivity distribution along x and y directions are shown in Fig. 2e. In this figure, it can be seen how the strain in y direction at 12.5% and 20% caused the increase of ρ_xx_ by 4.73% and 12.65%, as well as ρ_yy_ by 8.58% and 24.02%, respectively. These results imply that strains induce anisotropic resistivity change. From the literatures^55^,^62^, long and slender MWCNT networks tend to be aligned along the direction of applied strain. This structural evolution of the MWCNT networks can induce anisotropic resistivity.

### Fundamentals of anisotropic electrical impedance tomography

**Figure 3:**
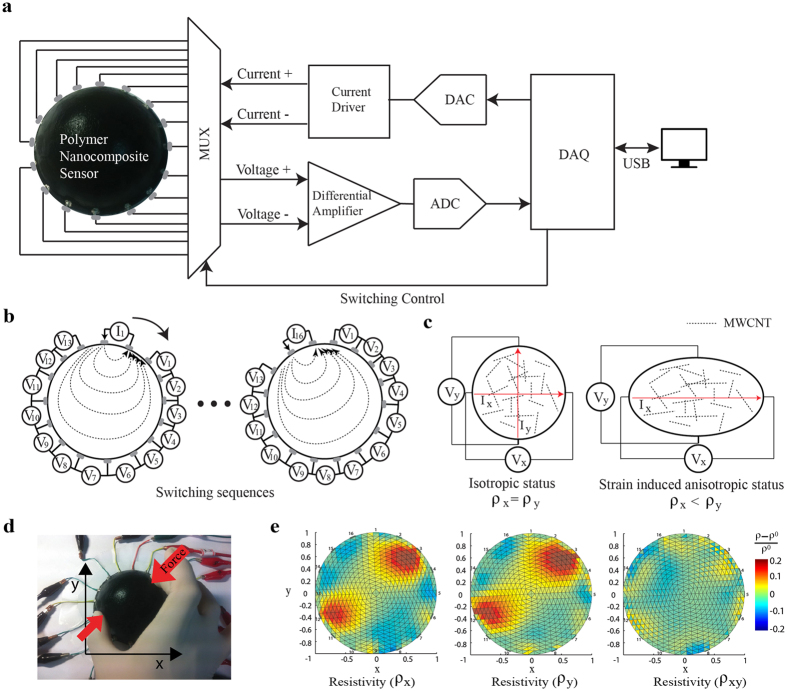
Schematic of electrical impedance tomography and concept of multi-dimensional strain measurements. (**a**) Schematic diagram of hardware configurations of electrical impedance tomography^53^. (**b**) Schematic of adjacent method injecting electrical current and measuring voltage potentials via multiplexing^53^. (**c**) Illustration of the isotropic status and strain induced anisotropic status. (**d**) Photograph of the test with two fingers and hemispherical shaped MWCNT-silicone elastomer nanocomposites. (**e**) Results of the test showing two normal resistivities and one shear resistivity.

As mentioned above, the anisotropic electrical impedance tomography is suggested in this work to calculate the internal resistivity distribution of soft and conductive medium without fabricating stretchable electrodes within the medium. We focused on the reconstruction of anisotropic resistivity distributions to evaluate the multi-directional strain distribution by using the anisotropic piezoresistive characteristics of the nanocomposite. Figure 3a shows the schematic diagram of the anisotropic resistivity reconstruction system. MWCNT-silicone elastomer nanocomposite sensor structure was connected to the electrical interconnections at its boundary with silver paste. The interconnections were connected to the multiplexer that switched four connections (current+, current−, voltage+, voltage−) controlled by a computer. As a switching sequence, adjacent method, which provides a symmetric switching sequence, was chosen to inject the electrical current all over the nanocomposites^53^. Figure 3b illustrates the method in which currents are injected through a pair of adjacent electrodes and electrical potentials are measured from the other adjacent electrodes. The current injection and voltage measurement were rotated through all adjacent electrode pairs to cover the entire domain. The anisotropic resistivity distributions were calculated from the two sets of the data which were measured in isotropic status and in strain induced anisotropic status as shown in Fig. 3c. The data in isotropic status was measured when the resistivity measured in x and y directions are the same due to the random orientation of the MWCNT fillers. After the strain is applied to the sensor, the anisotropic change of electrical resistivity is caused by the alignment of the long and slender MWCNTs^6^. The reconstruction of the anisotropic resistivity distributions was conducted by using inverse calculation (Supplementary Note 3). Three resistivity distributions are computed from the anisotropic resistivity reconstruction; two normal resistivity distributions and one shear resistivity distribution. Normal resistivity distribution is defined where the direction of the current injection and the direction of the voltage measurement are in parallel. Shear resistivity distribution is defined where the directions of the current injection and voltage measurement are perpendicular to each other. The hemispherical shaped strain sensor was used to identify contact locations and intensity from the test (Fig. 3d). From the resistivity reconstruction procedures, three resistivity distributions were computed (Fig. 3e). The normal resistivity showed increases at the locations where pinch forces were applied.

### Strain sensing performances

**Figure 4:**
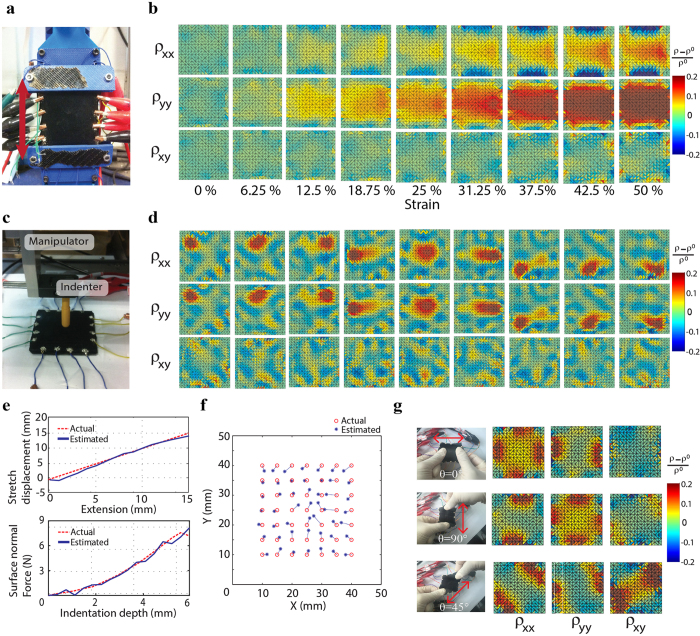
Strain sensing test of MWCNT-silicone elastomer nanocomposite strain sensor. (**a**) Photograph of stretching test using tensile testing machine and (**b**) results of anisotropic resistivity distributions in tensile test. (**c**) Photograph of surface compression test using 3 dimensional manipulator with an indenter and (**d**) results of anisotropic resistivity distributions in the indentation test. (**e**) Results of estimated stretch displacement from the tensile test and surface normal force from the indentation test. (**f**) Plot of estimated contact locations with respect to the 49 individual contact points. (**g**) Anisotropic resistivity distributions for stretching in three different directions.

Performances of the proposed sensor were measured by tensile test and indentation test using a square shaped (50 mm × 50 mm × 5 mm) planar sensor. Besides, tensile test was done using a custom-designed tensile testing machine (Fig. 4a). In this experiment, tensile strain was applied to the sensor from 0% to 50% by 16 intervals. Figure 4b shows the results of anisotropic resistivity distributions calculated by aEIT technique. From 0 to 50% strains, both normal resistivities in x and y directions were increased. The resistivity distribution along x direction (ρ_xx_) increased by 10% in average while the resistivity distribution along y direction (ρ_yy_) increased by 20% in average. This result implies that the sensor can distinguish the direction of the strain from the anisotropic resistivity distributions. Although the average value of the resistivity distribution along y direction showed an exponentially increasing curve due to the characteristic of the MWCNT-silicone elastomer nanocomposites as shown in Fig. 2c, exponential regression was used to estimate stretch displacement because the resistivity of the nanocomposite increased exponentially when stretched. The estimation error of the stretch displacement was 0.54 ± 0.53 mm and R^2^ value was 0.98. (Fig. 4e).

The surface indentation test was conducted using three dimensional manipulator with a sphere shaped indenter to evaluate the strain localization performance on the square shaped planar sensor (Fig. 4c). As shown in this figure, the sensor was located on the ground and the indenter pressed the sensor along the normal direction to the surface. Indented locations on the planar surface of the sensor were from 10 mm to 40 mm, with an interval of 5 mm in the x and y directions, resulting in 49 indented locations in total. Figure 4d shows the anisotropic resistivity distributions for 9 different contact locations. The results of the surface indentation test demonstrates that the compressive strain to the surface of the sensor induces similar resistivity change along x and y directions. To show the performances of the strain sensor, stretch displacement from the tensile test was estimated from the average of the change in resistivity distributions along y directions (ρ_yy_). The surface normal force in the indentation test was estimated by the average of the change in resistivity distribution along x and y directions. To evaluate the performances of surface normal force, indentation test was conducted as shown in Supplementary Figure 5. In the indentation test, the estimation error of the surface normal force was 0.61 ± 0.62 N (Fig. 4e). The minimum pressure the sensor can detect was 0.1727 N as evaluated from the limit of detection^63^ (see Supplementary Figure 6). The response time of the sensor was evaluated using chirp signal shaped compression. The cutoff frequency of the sensor system including data acquisition, computation of resistivity distribution was calculated as 2.13 Hz (Supplementary Figure 7). Although the response time of the sensor was not sufficiently fast, this can be improved by using stiffer elastomers since the slow response time seems to be due to the softness and viscoelasticity of the nanocomposite. The location of compressive contact was estimated by calculating the centroids of the area of normal resistivity distributions (Supplementary Note 4). Figure 4f displays the errors between the estimated and actual contact locations for 49 individual contact points. Although the errors of the estimation tend to be larger at locations farther from the boundary electrodes (i.e. central regions of the sensor) due to the imperfection of the resistivity reconstruction model, estimation error of the contact locations was 1.88 ± 0.95 mm (37.6 ± 19.0% for 5 mm spatial resolution).

Figure 4g illustrates the results of the stretching test with different orientations (θ = 0°, 45°, 90°) from the x axis to show the identification capability of stretch directions. When the sensor was stretched along the x axis (θ = 0°), the normal resistivity distribution in the x axis (ρ_xx_) increased. A stretch to the y axis (θ = 90°) showed an increase of normal resistivity distribution in the y axis (ρ_yy_). When the sensor was stretched by an angle of θ = 45°, shear resistivity distribution (ρ_xy_) increased. These results imply that the anisotropic EIT technique can identify the stretching direction.

### Demonstration of human-machine interface device

**Figure 5:**
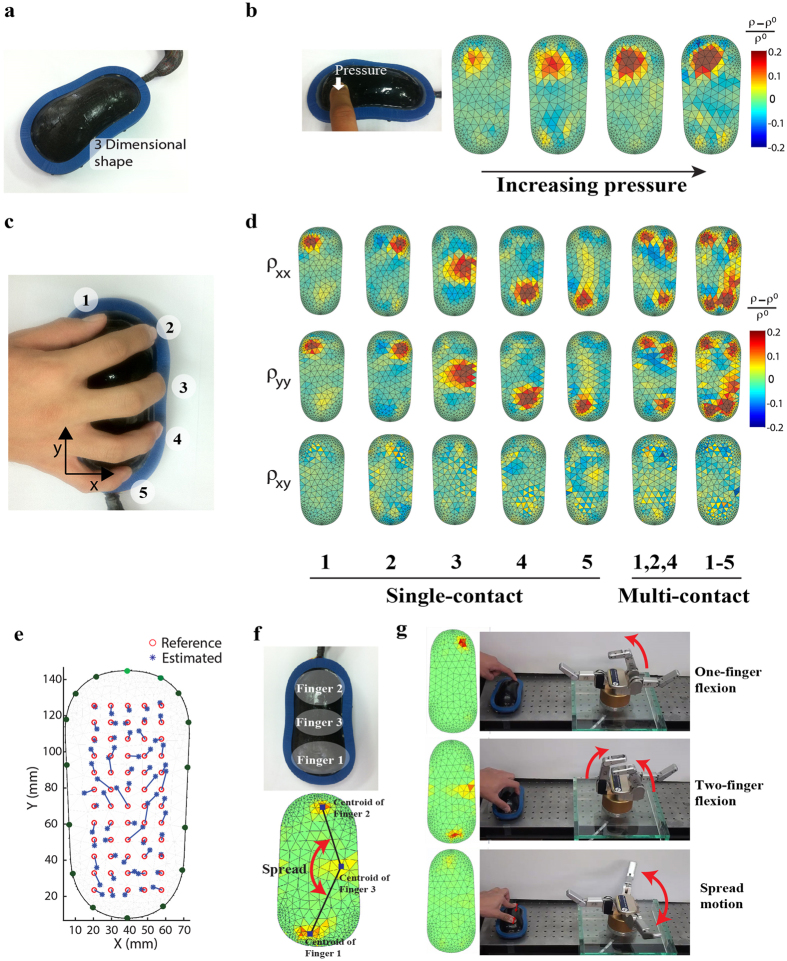
Application of MWCNT-silicone elastomer nanocomposite strain sensor to human machine interface. (**a**) Photograph of the developed three dimensional contoured sensor device. Experiments of (**b**) Contact pressure measurement test. (**c)** Predefined locations of five fingers for the experiment and (**d**) the results of single and multi-contact experiments. (**e**) Comparison of estimated and actual contact locations. (**f**) Illustration of divisions for three sections to control robot fingers and three centroid points. (**g**) Control of robotic finger motions by MWCNT-silicone elastomer nanocomposite strain sensor.

The proposed sensor has a great potential to be used as a soft human-machine interface because it can detect both contact force and location and be formed in various three dimensional shapes while covering a large area. Figure 5a represents an example of three dimensional contoured human-machine interface detecting contact pressures from five fingers. Figure 5b illustrates how the sensor could distinguish different pressure levels. When the finger applied pressure on the device surface, the electrical resistivity is locally increased at the location of pressure, as represented by the color intensity in the figure. Figure 5c shows the defined locations of five fingers chosen for the experiment. In Fig. 5d, the first five columns illustrate the distributions of resistivity change when each contact location is individually pressed. Meanwhile, the results of multi-contact tests in the last 2 columns exhibit the capability of our sensor in the detection of various multi-pressure patterns. The estimation accuracy of contact locations for the three dimensional contoured interface device was examined by the manipulator as shown in Fig. 4c. Indented locations on the device surface were from 20 mm to 60 mm with an interval of 10 mm in the x directions and from 25 mm to 125 mm with an interval of 10 mm in y directions, resulting in 60 indented locations in total (Fig. 5e). Estimation error of the locations was 4.80 ± 3.05 mm (48.0 ± 30.5% for 10 mm spatial resolution). This relatively large estimation error was caused by the discrepancy between the resistivity reconstruction model defined in two dimensions and the actual sensor shape with three dimensional surfaces. Due to this discrepancy, the contact locations on the device was projected to the two dimensional resistivity plane.

To demonstrate the potential of this human-machine interface device in the robotic applications, the signals from the device was utilized to control a robot hand (BH8-262, Barrett Technology, USA) that can bend and spread its fingers. Since this robot hand only has three fingers, the same number of control points were set in the sensor. In specific, the device was divided into three sections and each contact location was calculated (Fig. 5f). The average of the resistivity change from each section was used to control the rotation speed of each robotic finger. Besides, the spread angle calculated from the centroids of three contact points was used to control the spread angle of the robotic fingers. In detail, the robot fingers were spread if the spread angle was larger than 180 degrees and the difference of the angle was mapped to the spread speed of the robot finger (Fig. 5f,g). The control of the robot hand was performed within 0.1 seconds of time interval including the time for data acquisition, calculation of resistivity distributions, and display of the resistivity distributions (Supplementary Movie 1).

## Discussion

This paper presented a multi-point, multi-directional strain distribution sensor using anisotropic electrical impedance tomography (aEIT) on MWCNT-silicone elastomer nanocomposite-based three dimensional strain sensor. Critical components to this work are the fabrication of nanocomposites with strain induced anisotropic resistivity and implementation of aEIT system to calculate the multi-directional resistivity distributions. The resistivity distributions are computed from the voltage potentials measured at the electrodes on the boundary of the nanocomposites. This approach could be used to realize multi-directional strain distribution sensors with large coverage area and three dimensional contoured shapes. A remaining technical challenge is the enhancement of anisotropic piezoresistivity of nanocomposites and calculation accuracy in the aEIT system. Besides, applying the aEIT technique to other piezoresistive materials such as liquid metal or nanowires composites will represent another promising direction for the future research.

## Methods

### Device fabrication

Conductive polymer composite should have piezo-resistivity to be used as a strain sensor. We used multi-walled carbon nanotube (MWCNT, Hyosung, South Korea) as a conductive filler and silicone rubber (EcoFlex0030, Smooth-On, Inc, USA) as a polymer matrix. Mixing process was as follows. Firstly, MWCNT and silicone rubber were mixed by planetary centrifugal mixer (PDM-300, EXAKT, Germany) for 2 minutes. Then mixed composite was dispersed by three roll mills (80E, EXAKT, Germany) in four stages. Different mixing ratios of MWCNT and silicone rubber was used (2.5 wt%, 3.5 wt%, 4.5 wt% and 5 wt%). Since the sensitivity of the piezo-resistivity mainly depends on the volume fraction of the MWCNT and polymer matrix, the volume fraction showing the highest sensitivity was chosen. Acrylonitrile butadiene styrene (ABS) based plastic molds were fabricated to various shapes using three dimensional printing machine (Dimension elite, Stratasys, USA). The plastic molds had tiny air gaps for ventilation. Surfaces of the plastic molds were covered by release agent (ER200, Mann release technologies, USA). In injection molding process, liquid dispenser (AD3000C, Iwashita Engineering, Japan) provided compressed air to pressed MWCNT-silicone elastomer composite. After the composite filled the plastic mold, an oven (OF-02, Jeiotech, South Korea) cured the composite. To attach electrodes to the composite, electrical wires were fixed using silver paste (ELCOAT, CANS, Japan) to provide stable electrical contact condition.

### Device characterization

The surface morphology of MWCNT-silicone rubber was characterized by field emission scanning electron microscopy (FE-SEM, Sirion, FEI, USA) operating at an acceleration voltage of 10 kV. To prepare the sample for SEM imaging, specimens were fabricated to have minimum thickness. After the specimen is located on the carbon tape, thin (~10 nm) platinum layer was sputtered. For the measurement of current-voltage (I-V) characteristics, silver paste was applied to the opposite sides of MWCNT-silicone rubber in order to reduce the contact resistance. The I-V curves were obtained by semiconductor parameter analyzer (4155A, HP, USA). To characterize anisotropic resistivity, 4 point probe (4-point meter, Dasol engineering, South Korea) was used. In order to measure the mechanical responses of the nanocomposites, a custom-designed tensile machine containing a computer-controlled linear guided actuator and a force sensor was utilized. In order to acquire the data and to control the aEIT system, data acquisition system (cDAQ 9174, National Instruments, USA) was operated with a sampling frequency of 30 kHz. The resistance of the specimen was measured simultaneously using constant current source and voltage meter from the each end. A differential voltage to current converter circuit was implemented to provide electrical currents to the sensor using operational amplifiers (OPA 177, Texas instruments, USA). The voltage difference was measured by a differential amplifier (INA 128, Texas instruments, USA).

## Additional Information

**How to cite this article**: Lee, H. *et al*. Soft Nanocomposite Based Multi-point, Multi-directional Strain Mapping Sensor Using Anisotropic Electrical Impedance Tomography. *Sci. Rep.* **7**, 39837; doi: 10.1038/srep39837 (2017).

**Publisher's note:** Springer Nature remains neutral with regard to jurisdictional claims in published maps and institutional affiliations.

## Supplementary Material

Supplementary Figures and Notes

Supplementary Video
