# Supplementary material for: Soft Nanocomposite Based Multi-point, Multi-directional Strain Mapping Sensor Using Anisotropic Electrical Impedance Tomography
Source: Sci Rep. 2017 Jan 25;7:39837. doi: 10.1038/srep39837 (PMC5264388; doi:10.1038/srep39837)
Supplement: Supplementary Figures and Notes [file srep39837-s1.pdf]

Soft Nanocomposite Based Multi-point, Multi-directional Strain Mapping Sensor Using Anisotropic Electrical Impedance Tomography

AUTHOR NAMES

*Hyosang Lee, Donguk Kwon, Haedo Cho, *Inkyu Park, *Jung Kim*

AFFILLIATIONS

Korea Advanced Institute of Science and Technology, 291 Daehak-ro, Yuseong-gu, Daejeon 305-701, Republic of Korea

CONTACT INFORMATION

inkyu@kaist.ac.kr, jungkim@kaist.ac.kr

Supplementary Information

Video 1: Demonstration video using the human-machine interface device and robot hand. The interface device was used to calculate resistivity distribution and control robot hand for single-finger flexion, two-finger flexion, three-finger flexion, spread motion, and object manipulation.

Supplementary Figures

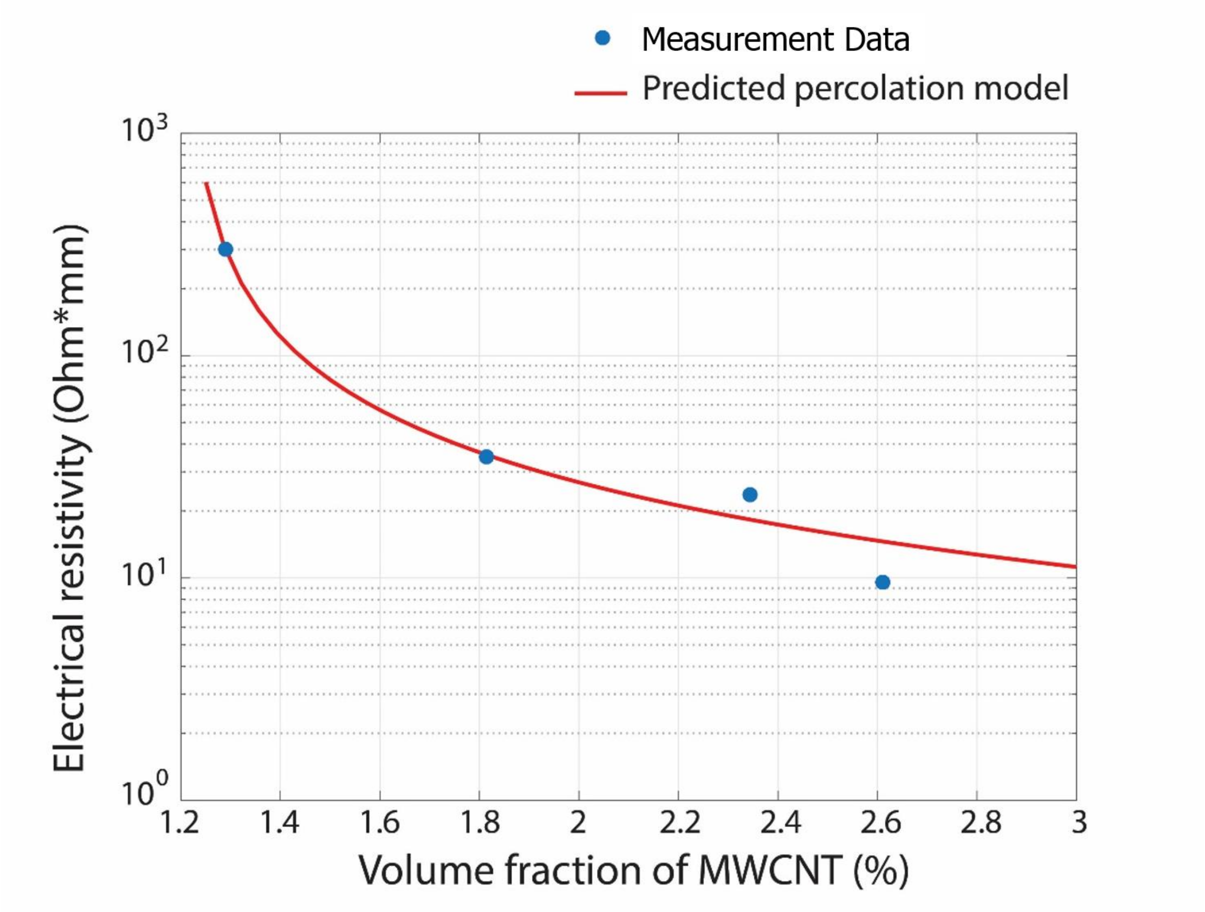

Supplementary Figure 1. Resistivity of the specimens with four different volume fractions of MWCNT (1.29(2.5), 1.81(3.5), 2.34(4.5), and 2.61(5.0) vol%(wt%)). The graph shows comparison with the predicted values calculated by the percolation model (Supplementary Note 1).

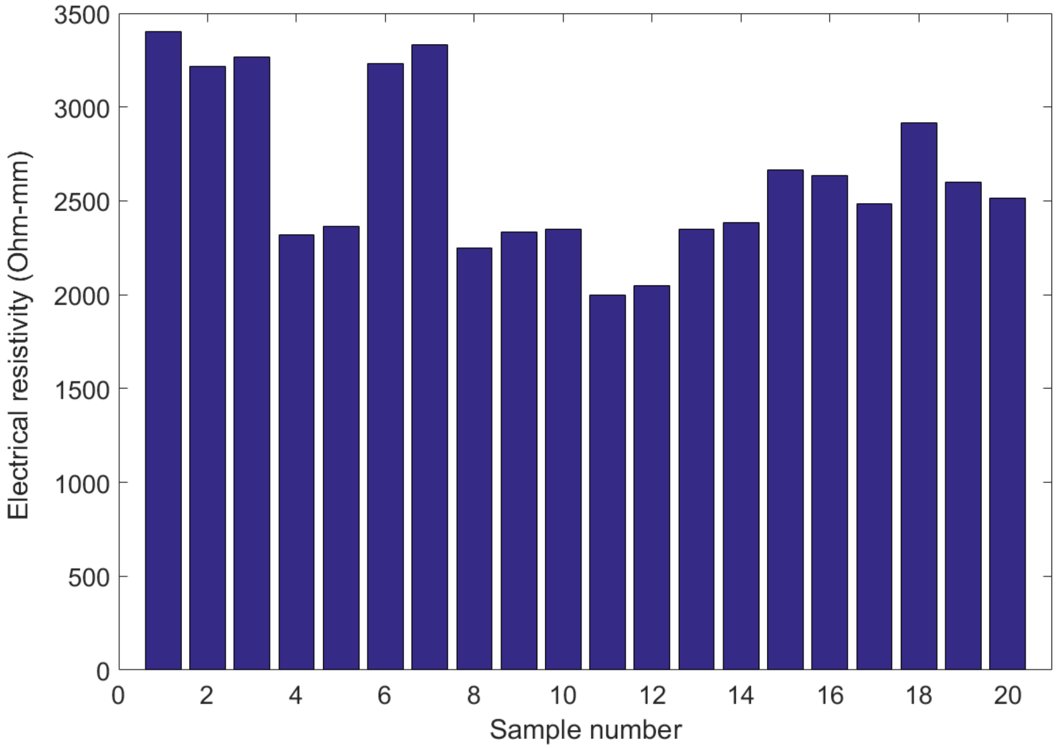

Supplementary Figure 2. Electrical resistivity of the samples with rectangular parallelepiped shape (length=3 mm, depth=10 mm, and width=5 mm) and 1.29 vol %. The electrical resistivity was measured by resistance meter (179 RMS multimeter, Fluke, USA). Mean and standard deviation of the resistivity of 20 samples were 2633 ± 439 ohm-mm.

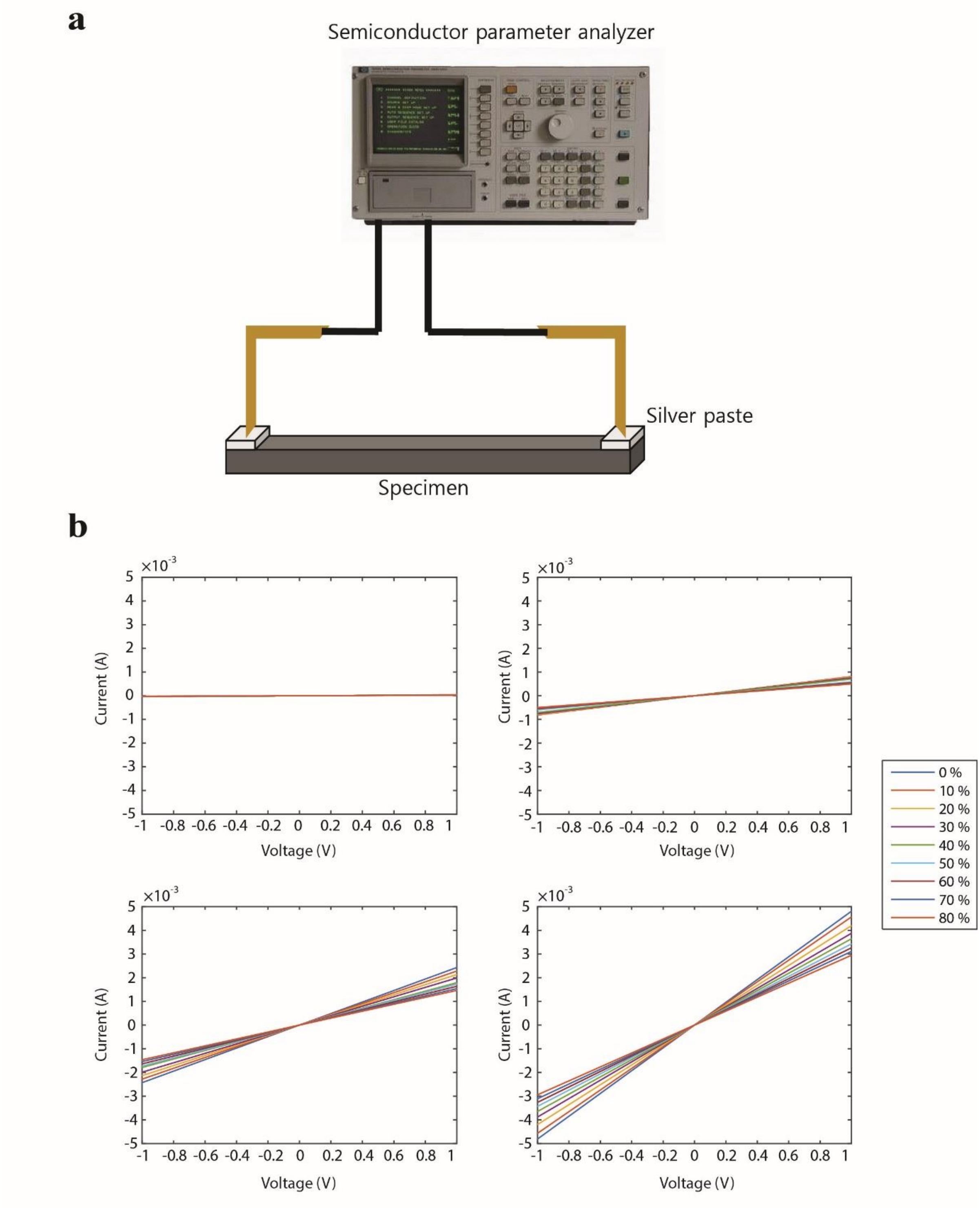

Supplementary Figure 3. Voltage-current (I-V) relationship of the specimens. (a) Schematic diagram of testbed including semiconductor parameter analyzer (4155A, HP, USA) and specimen with silver paste electrodes. (b) Graphs of the relationship between voltages and currents under extension (0% to 80% strain). The result implies that the MWCNT-silicone elastomer nanocomposites exhibit ohmic behavior.

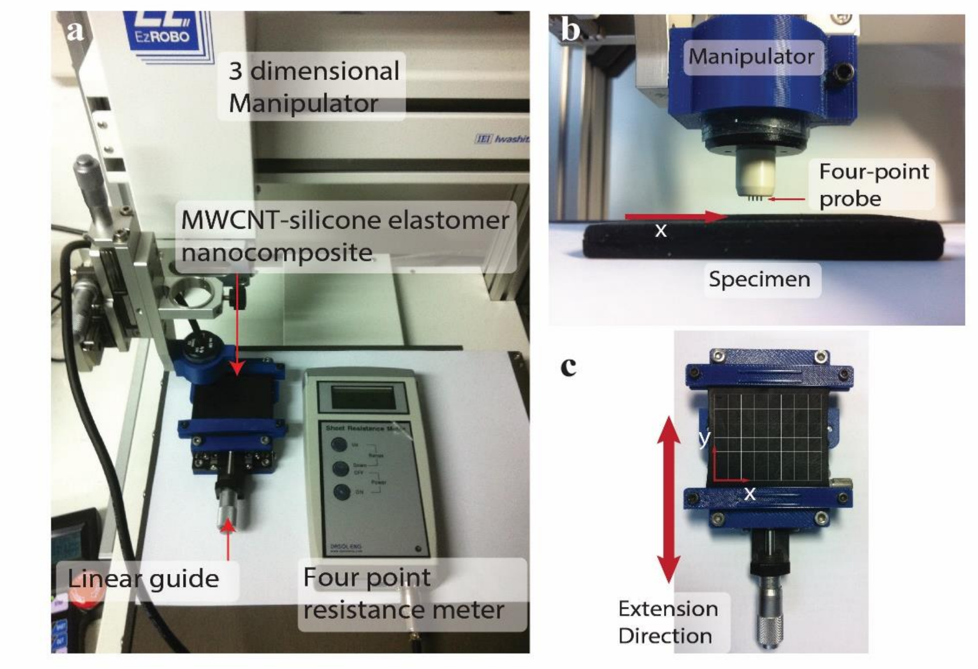

Supplementary Figure 4. Photographs of experimental setup to measure the anisotropic surface resistance of MWCNT-silicone elastomer nanocomposite. (a) Setup for three dimensional manipulator and (b) alignment of four-point probe to x direction and manipulator. (c) Apparatus to exert tensile strain to the planar shaped specimen along y direction. Anisotropic resistivity was measured from the literature (Supplementary Note 2)

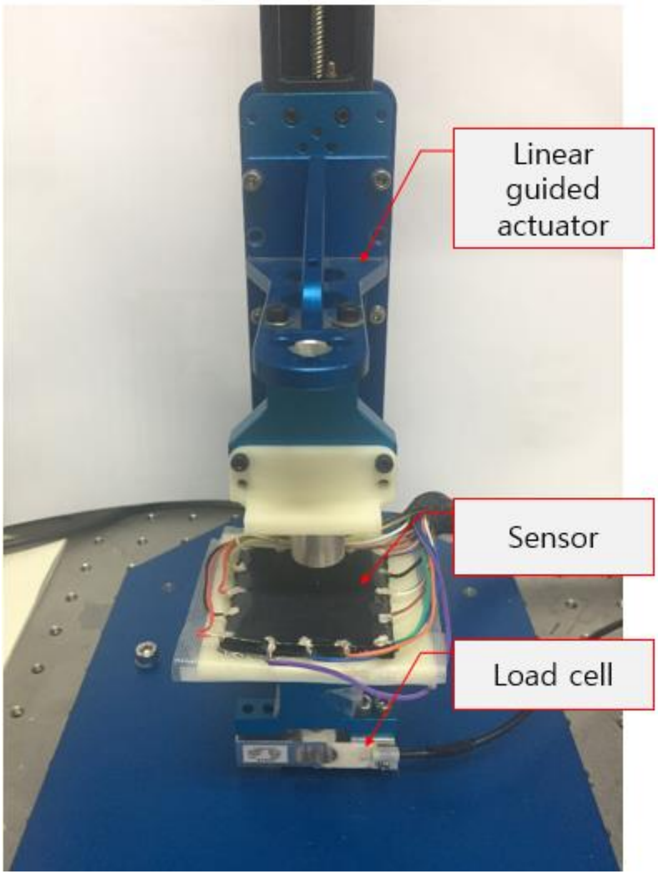

Supplementary Figure 5. Photographs of indentation test setup to measure the performances of surface normal force estimation.

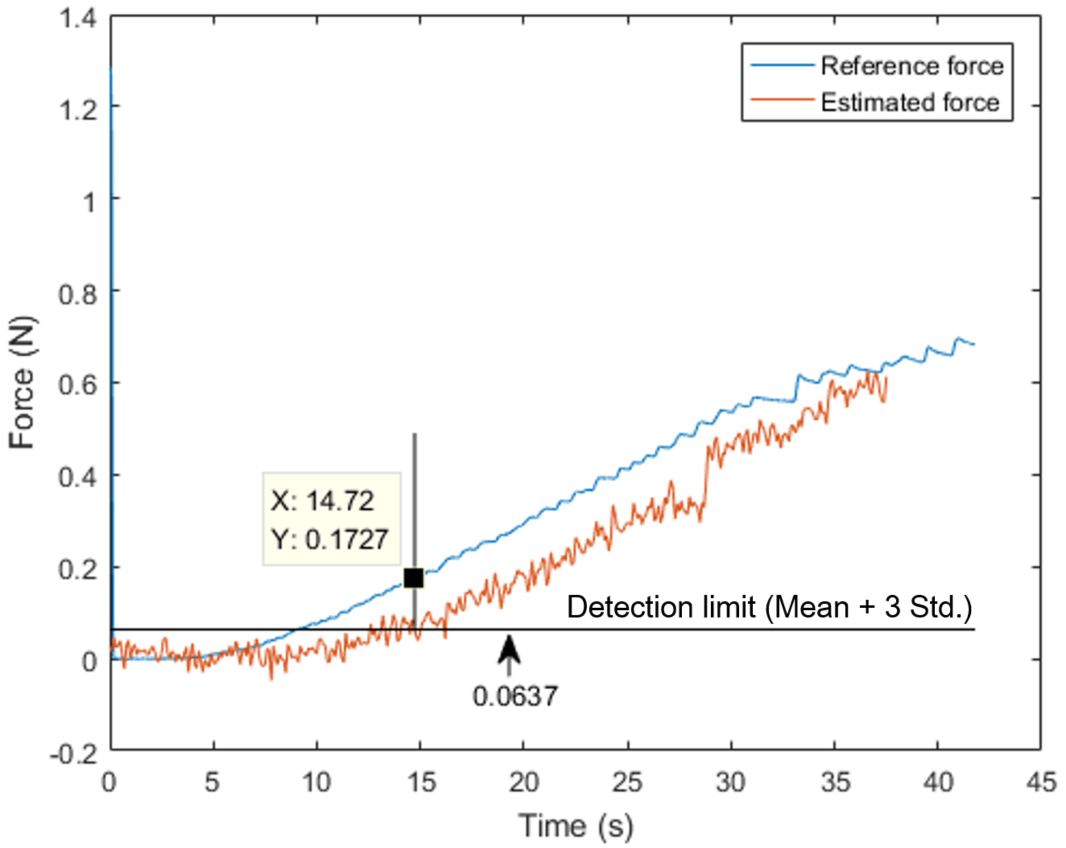

Supplementary Figure 6. Experimental result of indentation test to measure the pressure that the sensor can detect. Detection limit of the estimated force was computed from the mean and standard deviation of the estimated force when pressure is not applied to the sensor.

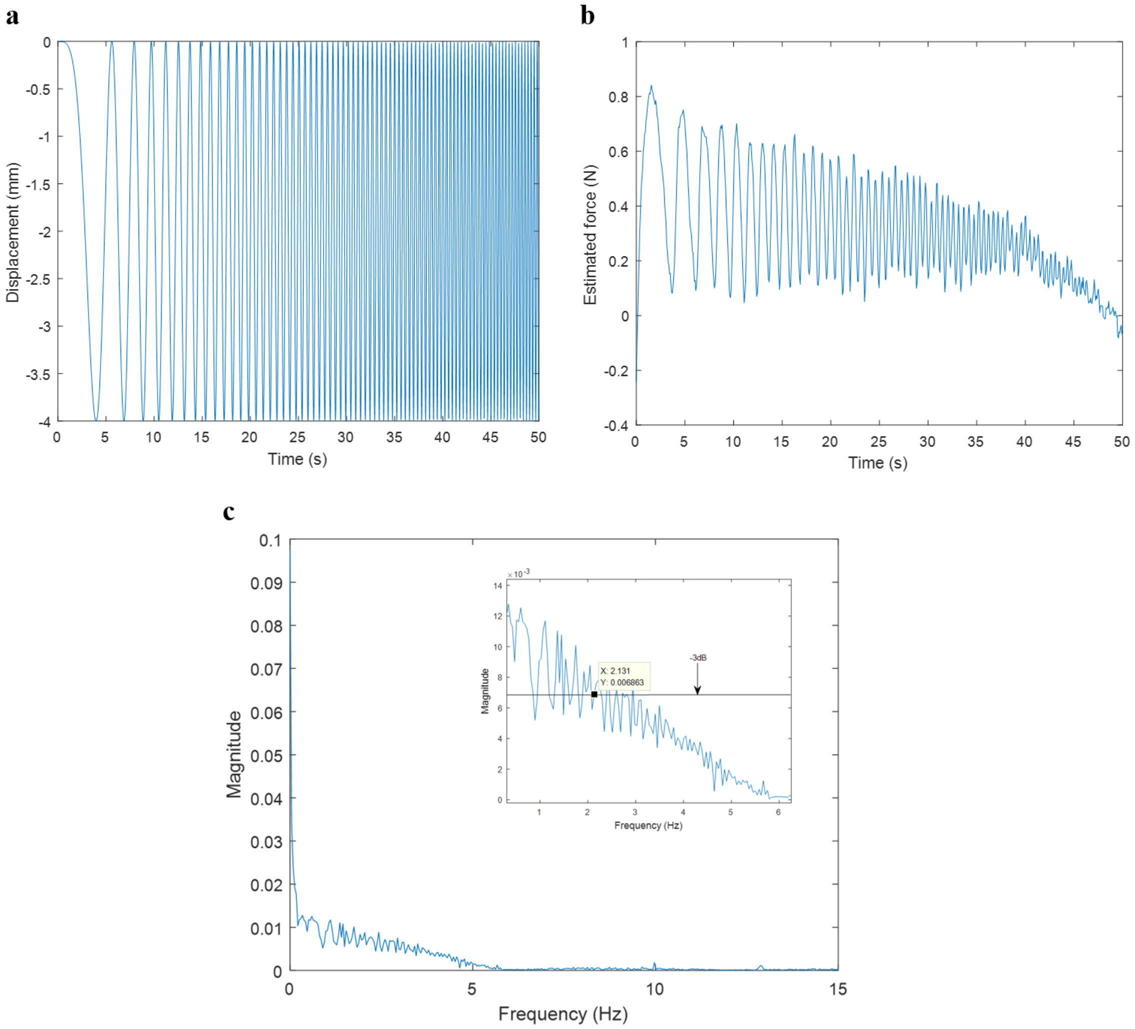

Supplementary Figure 7. Experimental results of dynamic indentation test. (**a**) Results of chirp signal shaped displacement having frequency of from 0 Hz to 10 Hz. (**b**) Results of estimated force during dynamic indentation test. (**c**) Results of fast Fourier transform (FFT) of the estimated force.

Supplementary Notes

Supplementary Note 1. Consistency with percolation model

The electrical conductivity of the conductive polymer composites can be expressed by percolation theory[1]. To evaluate the percolation threshold of the MWNCT/polymer nanocomposites, mathematical model of the percolation theory was adopted.

Mathematical representation of the percolation model is shown below.

$$\sigma = \sigma_i \left(\frac{\phi - \phi_c}{1 - \phi} \right)^{-t} \quad (1)$$

where σ, σ_i represents electrical conductivity of the composites and conductivity of the filler, respectively. ϕ represents volume fraction of MWCNT, and ϕ_c represents volume fraction of percolation threshold. t represents critical exponent.

From the Supplementary Figure 1, we have four different resistivity at each weight percentage of MWCNT. Three unknown parameters of the percolation model (σ_i, ϕ_c, t) were estimated using nonlinear least square method.

$$f(\sigma_i, \phi_c, t) = 0 \quad (2)$$

σ_i was determined as 6.1463 S/mm which means the conductivity of MWCNT. ϕ_c was determined as 0.0121 which means percolation threshold of the MWCNT-silicone elastomer nanocomposite is 1.21 vol%. t was determined as 1.06. From the previous study[2], the percolation threshold was 1.2 vol% for silicone rubber and MWCNT[3] which is quite consistent. Critical exponent is expected to depend on the dimensionality of the system and is close to 1.33 in two dimensions following to theoretical simulation for randomly oriented cylindrical fillers [1]. This result is also consistent with the estimated result because specimens were fabricated into flat.

Supplementary Note 2. Anisotropic resistivity measurement using inline four-point probe

Evaluation of the electrical resistivity in the case of an anisotropic solid is possible by using in-line four-point probe for 3D bulk medium [4]. Anisotropic resistivity consists of three principle resistivity (ρ_x, ρ_y, ρ_z). When the pins of the four-point probe are aligned to the direction of x axes, resistance measured in x direction (R_x) can be represented as below.

$$R_x = \frac{V_x}{I_x} = \frac{1}{2\pi s}\sqrt{\rho_y \rho_z} \quad (1)$$

where *s* is distance between the pins of the probe.

This means resistance measured in x direction from 3D bulk specimen is the geometric mean of the resistivity components along the other two principal axes. As shown in the Supplementary Figure 4 (b), the specimen was thick enough to be considered as 3D bulk specimen. When the specimen was stretched in the x direction, the change of the resistance measured in x direction can be represented as follows.

$$\begin{aligned} R_x &= \frac{V_x}{I_x} = \frac{1}{2\pi s}\sqrt{\rho_y \rho_z} \\ \Delta R_x &= R_x - R_x^0 = \frac{1}{2\pi s}\sqrt{\rho_y \rho_z} - \frac{1}{2\pi s}\sqrt{\rho_y^0 \rho_z^0} \end{aligned} \quad (2)$$

Compared to the resistivity of y-direction (ρ_y), the resistivity change of z-direction (ρ_z) can be considered small.

Finally, the change of resistance in x direction can be represented as follows.

$$\Delta R_x = \frac{1}{2\pi s}\sqrt{\rho_z}\left(\sqrt{\rho_y} - \sqrt{\rho_y^0}\right) \quad (3)$$

Similarly, the change of resistance in y direction is related to the change of resistivity in x direction.

Supplementary Note 3. Implementation of anisotropic electrical impedance tomography

Electrical impedance tomography (EIT) is a resistance reconstruction technique used to estimate the internal conductivity of a conductive medium using measurements from the boundary of the medium[5]. Anisotropic EIT systems have been formulated by a few researchers[6–8].

Voltage potentials across the conductive continuum medium can be modeled using Kirchoff's law.

$$\nabla \cdot \sigma \nabla \phi = 0 \tag{1}$$

where σ is conductivity which is a function of the spatial variable and φ is the scalar potential. Current injection can be modeled as a boundary condition.

$$j\,|_{\partial\Omega} = \sigma \nabla \phi \cdot n \tag{2}$$

where j is current density and n is normal vector from the surface of the boundary.

To solve the equations (1) and (2) for the given geometry of the body, finite element method (FEM) was used to transform the continuous form of the problem into a discrete approximation problem [9]. Normally, main interest of the EIT system is to find the changes of the conductivity distribution. Difference imaging was proposed to achieve faster and more accurate results by reducing problems with unknown contact resistance, electrode positions. Difference imaging uses two sets of voltage potential measurement points where the change of the conductivity occurs. The differences of the voltage potentials can be modeled as below.

$$\partial V \approx J \partial \sigma + w \quad with\ known\ \sigma_0 \tag{3}$$

where ∂V is a difference in voltage potentials between two measurements and *J* is the Jacobian between changes in boundary potential and internal conductivity. Also, *w* is a noise vector and σ_0 is the initial conductivity.

Jacobian matrix can be calculated directly from the perturbation of each element of the FEM mesh and changes of potential at the electrodes.

$$J_{i,j} \approx \frac{\partial V_{dm}}{\partial \sigma_{ij}} = -\int_{voxel,k} \partial_i u(I^d) \bullet \partial_j u(I^m) dV \quad , i = 1,...,N,\ j = 1,...,K \tag{4}$$

where *d*, *m* are indices for current drive and voltage measurement. Also, *i* and *j* are indices for the coordinates. *u(I)* is the voltage potential of the internal domain.

From the equation (4), anisotropic resistance distribution ($\partial\sigma_{ij}$) can be reconstructed by using inverse calculation. Normally regularization methods are used because the inverse calculation of the Jacobian matrix is ill-posed.

$$\partial\sigma = J^{-1}\partial V$$
$$\partial\sigma = (J^T J + \alpha R)^{-1} J^T \partial V, \quad (regularization) \tag{5}$$

where α is a scalar value to control the amount of regularization and R is a regularization matrix that stabilize the inverse computation.

Supplementary Note 4. Calculation of resistivity centroid

From the indentation tests, contact location was determined from the resistivity distribution as shown in the figure below. The contact location was estimated by calculating centroid of resistivity changes as shown in equations (6).

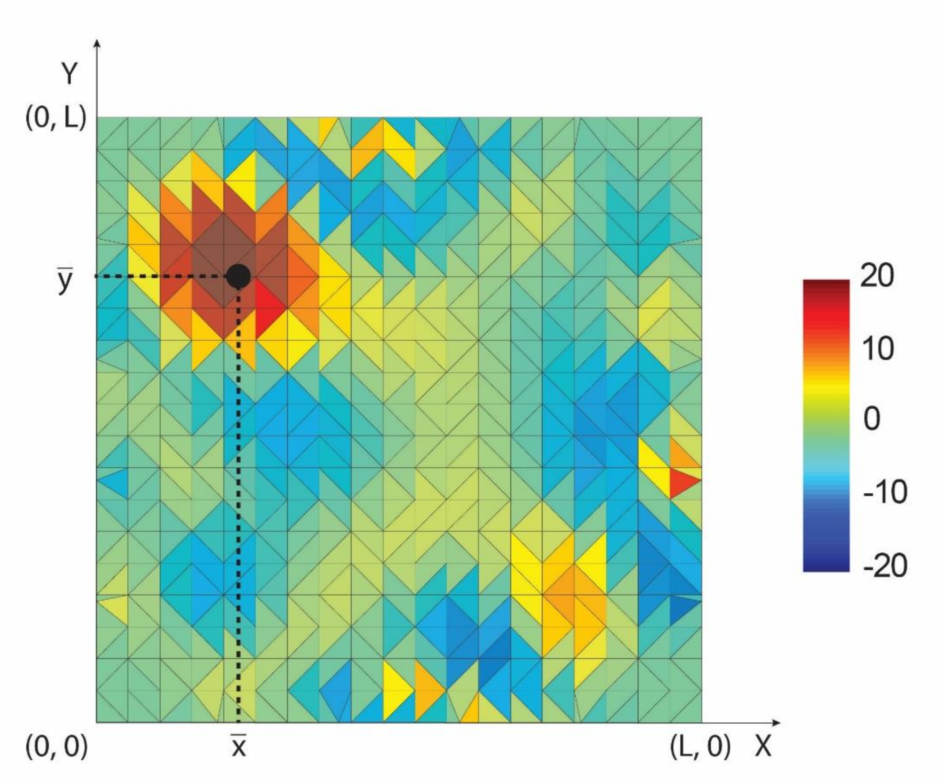

$$\overline{x} = \frac{\int_0^L x \bullet f(x,y)\,dx}{\int_0^L f(x,y)\,dx}$$
$$\overline{y} = \frac{\int_0^L y \bullet f(x,y)\,dy}{\int_0^L f(x,y)\,dy} \tag{6}$$

Supplementary References

[1] D. Stauffer and A. Aharony, *Introduction to percolation theory*. CRC press, 1994.

[2] W. Bauhofer and J. Z. Kovacs, “A review and analysis of electrical percolation in carbon nanotube polymer composites,” *Composites Science and Technology*, vol. 69, no. 10, pp. 1486–1498, 2009.

[3] M.-J. Jiang, Z.-M. Dang, and H.-P. Xu, “Giant dielectric constant and resistance-pressure sensitivity in carbon nanotubes/rubber nanocomposites with low percolation threshold,” *Applied physics letters*, vol. 90, no. 4, p. 042914, 2007.

[4] I. Miccoli, F. Edler, H. Pfnür, and C. Tegenkamp, “The 100th anniversary of the four-point probe technique: the role of probe geometries in isotropic and anisotropic systems,” *Journal of Physics: Condensed Matter*, vol. 27, no. 22, p. 223201, 2015.

[5] D. S. Holder, *Electrical impedance tomography: methods, history and applications*. CRC Press, 2004.

[6] J.-F. P. Abascal, S. R. Arridge, D. Atkinson, R. Horesh, L. Fabrizi, M. De Lucia, L. Horesh, R. H. Bayford, and D. S. Holder, “Use of anisotropic modelling in electrical impedance tomography; description of method and preliminary assessment of utility in imaging brain function in the adult human head,” *Neuroimage*, vol. 43, no. 2, pp. 258–268, 2008.

[7] J.-F. P. Abascal, W. R. Lionheart, S. R. Arridge, M. Schweiger, D. Atkinson, and D. S. Holder, “Electrical impedance tomography in anisotropic media with known eigenvectors,” *Inverse problems*, vol. 27, no. 6, p. 065004, 2011.

[8] C. C. Pain, J. V. Herwanger, J. H. Saunders, M. H. Worthington, and C. R. de Oliveira, “Anisotropic resistivity inversion,” *Inverse Problems*, vol. 19, no. 5, p. 1081, 2003.

[9] M. Soleimani, C. Gómez-Laberge, and A. Adler, “Imaging of conductivity changes and electrode movement in EIT,” *Physiological measurement*, vol. 27, no. 5, p. S103, 2006.
